# Empathy and the Underlying Psychosocial Basis of Aggressive Behavior and Sexual Trauma

**DOI:** 10.1192/j.eurpsy.2022.838

**Published:** 2022-09-01

**Authors:** M. Hazeghazam

**Affiliations:** Jesse Brown VAMC, Department Of Psychiatry, Chicago, United States of America

**Keywords:** Sexual trauma, Empathy, Power, Me too movement

## Abstract

**Introduction:**

The reports on the adverse impact of sexual trauma on mental health are known to the medical community. In workplaces where power hierarchy is an essence for the establishment, like the military, there has been a tsunami of reports on sexual trauma. Empathy plays a defining role in human relationships and development.

**Objectives:**

To explore the relationship between power and empathy by studying the prevalence of sexual assault among our population of women veterans who report their sexual assault occurred by a higher rank serviceman.

**Methods:**

A retrospective chart review was conducted in Women Health Clinic over 11 months period. A total of 117 charts were reviewed from 03/2019 to 02/2020. The information of 42 patients with sexual trauma was tabulated on an excel spreadsheet.

**Results:**

25 (59%) of 42 patients had military sexual trauma (MST). Of 25 who reported MST, the majority, 17 (72%), said trauma was from a serviceman with a higher status, and 2 (8%) were from the same rank.The distribution of military divisions was 14 (56%) army, 5 (20) % navy, and 4 (16%) were from the air force.

**Conclusions:**

Correlation between the prevalence of assaultive behavior and a higher status in rank was demonstrated in a sample of women veterans. 72% reported the higher rank servicemen caused the sexual offence. Our finding supports that a higher position in status is likely a determining factor for aggressive behavior. There is an opportunity to turn our attention to education and staff training to help them improve their compassion and empathy.

**Disclosure:**

No significant relationships.

